# The Risks of Exfoliative Esophagitis in Patients with Atrial Fibrillation: A retrospective observational study

**DOI:** 10.1097/MD.0000000000021681

**Published:** 2020-08-14

**Authors:** Hiroki Tajima, Toshiaki Narasaka, Daisuke Akutsu, Hideo Suzuki, Hirofumi Matsui, Kazushi Maruo, Hiro Yamasaki, Yuji Mizokami

**Affiliations:** a Department of Gastroenterology; b Department of Biostatistics; c Department of Cardiology, Faculty of Medicine, University of Tsukuba, Tsukuba, Japan.

**Keywords:** esophagitis, antithrombotic drug, obesity

## Abstract

The aging of the population has resulted in atrial fibrillation (AF) becoming increasingly prevalent. Treatment focuses on the prevention of thromboembolism through the use of catheter ablation or drug therapy with anticoagulants, such as warfarin or direct oral anticoagulants (DOACs). Dabigatran-induced exfoliative esophagitis has been reported as a rare side effect of DOACs. Although most cases are mild, some result in severe outcomes. However, the etiology of exfoliative esophagitis remains incompletely understood. The aim of this study is to investigate the etiology of exfoliative esophagitis and identify its risk factors by observational study.

The participants were 524 patients using anticoagulants who received catheter ablation for AF and subsequently underwent upper gastrointestinal endoscopy at University of Tsukuba Hospital. Exfoliative esophagitis was noted in 21 (4.0%) patients. Potential risk factors for exfoliative esophagitis were examined retrospectively by comparing patients with and without this condition across the following parameters that were extracted retrospectively from the electronic medical records: physical characteristics, comorbidities, blood-based cardiac markers, echocardiographic and endoscopic findings, and current medications.

Regarding physical characteristics, patients with exfoliative esophagitis had significantly higher body weight and BMI. No association was observed between exfoliative esophagitis and comorbidities. Associations were also not found for cardiac markers, echocardiographic findings, or endoscopic findings. In terms of current medications, patients receiving oral dabigatran showed the highest prevalence of exfoliative esophagitis at 8.8% (13/148). The adjusted odds ratio of dabigatran for exfoliative esophagitis was 10.3 by multivariable logistic regression analysis.

Obesity and oral dabigatran were found to be significant risk factors for exfoliative esophagitis.

## 1 Introduction

The aging of the population has resulted in atrial fibrillation (AF) becoming increasingly prevalent.^[1,2]^ In the United States, 0.89% of the population are reported to suffer from it, at a prevalence of 2.3% in adults aged 40 years, rising to 5.9% in people aged 65 years and 10% in people aged 80 years and older.^[3]^ An epidemiological survey of the Japanese Circulation Society showed that the prevalence was 3.44% for men, 1.12% for women in their 70s and 4.43% for men of that age, and 2.19% for women in their 80s and older.^[4]^ In patients with AF, thromboembolic events such as cerebral infarction are relatively common complications, which can be effectively prevented through the administration of oral anticoagulants. Direct oral anticoagulants (DOACs), such as dabigatran, are increasingly being administered and are reported to account for up to 62% of new anticoagulant prescriptions.^[5]^ DOACs are fast-acting and require no prothrombin time monitoring.^[6,7]^ They also enable patients to avoid the dietary restriction and drug combination risks required by warfarin. However, cases of dabigatran-induced exfoliative esophagitis and esophageal ulcers are increasingly being reported, with a recent study reporting an incidence of 20.9%.^[8,9]^ Most cases of exfoliative esophagitis involve mild symptoms such as heartburn and chest discomfort.^[10]^ However, more severe cases involving bleeding and extensive ulceration have also been reported.^[11]^

We have performed routine upper gastrointestinal endoscopies for AF patients following catheter ablations because esophageal mucosal injury following catheter ablation has been reported,^[12–14]^ and we have also encountered some cases of exfoliative esophagitis. In the present study, we aimed to clarify the prevalence and characteristics of exfoliative esophagitis in AF patients with anticoagulants and identify the risk factors.

## 2 Methods

**Figure 1:**
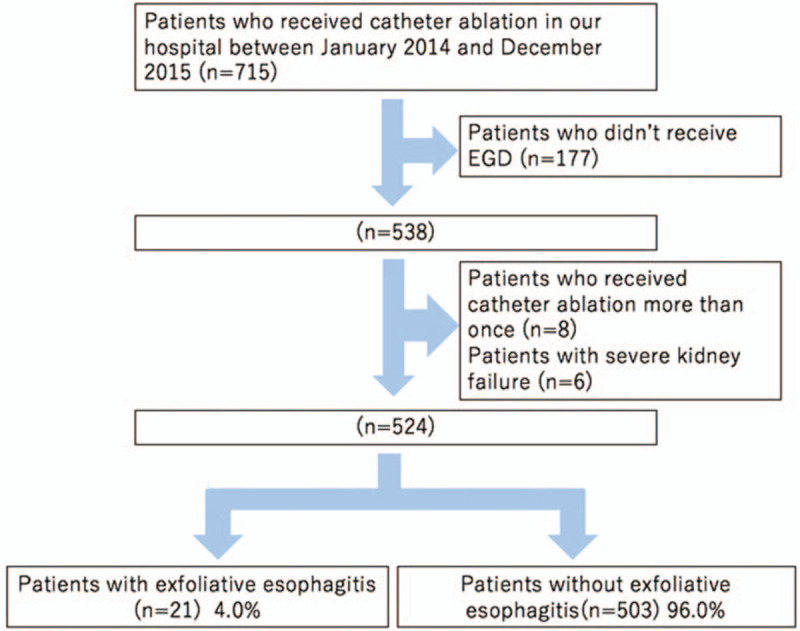
Enrollment in this study. A total of 715 patients received catheter ablation for AF; 177 patients without subsequent upper gastrointestinal endoscopy, 8 duplicate patients, and 6 patients with end-stage renal failure were excluded. The remaining 524 patients were divided into groups with (n = 21) and without exfoliative esophagitis (n = 503). AF = atrial fibrillation.

**Figure 2:**
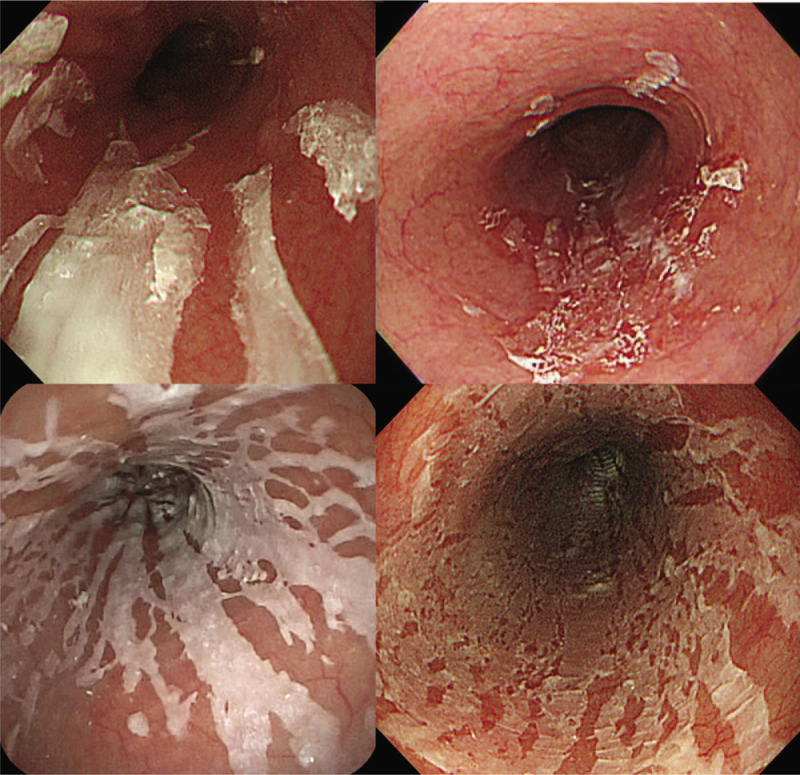
Endoscopic image of exfoliative esophagitis. Vertically oriented white strips of sloughing epithelium were characteristic endoscopic findings.

The participants in this study were AF patients hospitalized at the University of Tsukuba Hospital between January 2014 and December 2015. The inclusion criteria were patients using oral anticoagulants who underwent upper gastrointestinal endoscopy after catheter ablation. The exclusion criteria were the patients with upper gastrointestinal endoscopy after 29 days of the procedure and the patients with end-stage renal failure. During that period, 715 patients received catheter ablation for AF, 538 of whom subsequently underwent upper gastrointestinal endoscopy within 28 days after the procedure. After excluding duplicates (n = 8) and patients with end-stage renal failure (n = 6), 524 patients were included in the study (Fig. 1). Exfoliative esophagitis was defined as esophagitis involving vertically oriented white strips of sloughing epithelium (Fig. 2) and was observed in 21 (4.0%) patients. The following variables were extracted retrospectively from the electronic medical records in 2 patient groups, namely, those with or without exfoliative esophagitis: clinical characteristics (age, sex, height, body weight, body mass index [BMI]), comorbidities (hypertension, diabetes mellitus, coronary artery disease, valvular heart disease, cardiomyopathy), echocardiographic findings (left atrial diameter [LAD], left atrial volume [LAV], left atrial volume index [LAVI], left ventricular ejection fraction), serum cardiac markers (troponin T, brain natriuretic peptide [BNP], N-terminal pro-B-type natriuretic peptide [NT-proBNP], human atrial natriuretic peptide [hANP]), number of days after catheter ablation to endoscopy, endoscopic findings (hiatal hernia, reflux esophagitis), and kinds of anticoagulants (apixaban, dabigatran, edoxaban, rivaroxaban, warfarin). Potential risk factors for exfoliative esophagitis were examined retrospectively by performing the following statistical analyses.

To avoid detection bias, rigorous statistical analysis was conducted with experts in biostatistics. Diagnostic performance was evaluated using SPSS version 25.0 software and R statistical environment software. First, we conducted the Shapiro-Wilk test to investigate which variables were not normally distributed among the clinical characteristics, cardiac ultrasonography findings, and heart-related markers in blood biochemical tests. Second, we carried out Student *t* test for those with a normal distribution and the Mann–Whitney test for those without one. Regarding medications and endoscopic findings, we conducted the *χ*^2^ test. To detect the prognostic risk factors for exfoliative esophagitis, a multiple logistic regression model including the following candidate prognostic factors was applied: BMI, LAD, LAV, LAVI, log(pre BNP), log(pre NT − proBNP), log(pre hANP), sliding herniation, and coronary artery disease, where the variable selections were conducted using a stepwise procedure with a threshold determining inclusion or exclusion of *P* = .15. The goodness-of-fit for the logistic model was evaluated with the c-statistic and the Hosmer-Lemeshow test. Additionally, we conducted multiple logistic regression analysis and analyzed the risk contributed by each factor upon adjusting for sex and age. All results were considered statistically significant when *P* values were <0.05.

The present study was approved by the University of Tsukuba Hospital Institutional Review Board (H28-57). An informed consent was obtained in the form of opt-out on the website in accordance with the World Medical Association Declaration of Helsinki.

## 3 Results

Although no differences in age, male-to-female ratio, or height were observed between patients with and without exfoliative esophagitis, statistically significant differences in body weight and BMI were observed between these patient groups. The mean body weight (72.3 vs 66.4) and mean BMI (25.8 vs 24.2) were statistically significantly higher in patients with exfoliative esophagitis. No significant differences in the prevalence of hypertension, diabetes mellitus, coronary artery disease, valvular heart disease, and cardiomyopathy were observed between the 2 groups. Statistically significant differences were also not observed between the groups in terms of echocardiographic findings. This includes comparisons of left atrial diameter, left atrial volume, left atrial volume index, and left ventricular ejection fraction. In terms of blood test results, no statistically significant differences were observed between the two groups for the levels of troponin T, BNP, NT-proBNP, or hANP either before or after the procedure. In addition, no statistically significant differences were observed in the number of days after catheter ablation to endoscopy and the prevalence of hiatal hernia or reflux esophagitis (Table 1).

**Table 1:**
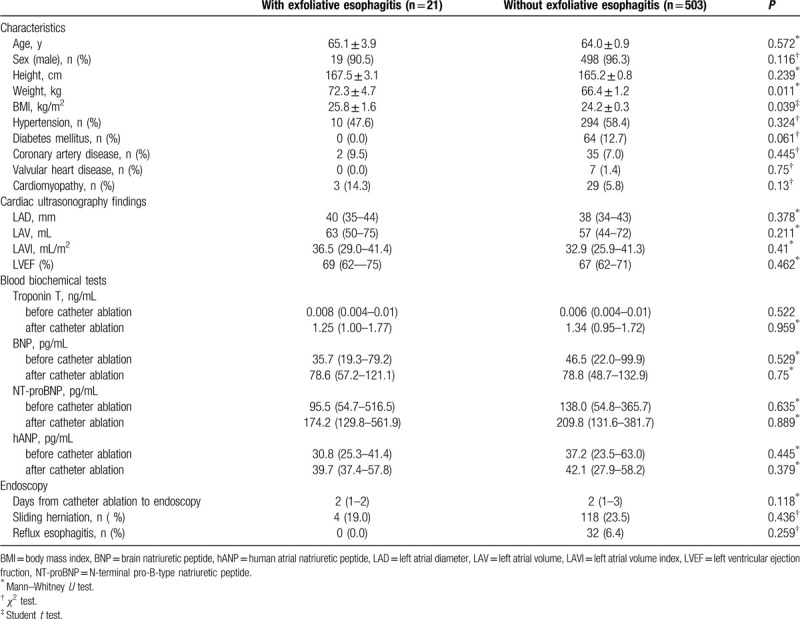
Characteristics, cardiac function and serum biochemical tests and endoscopic related findings with or without exfolative esophagitis.

In terms of anticoagulants, the prevalence of exfoliative esophagitis was the highest for patients who received oral dabigatran, at 8.8% (13/148). This was followed by patients who received edoxaban (6.3%, 1/16), warfarin (3.1%, 4/131), rivaroxaban (1.6%, 2/123), and apixaban (the lowest at 0.9%, 1/106) (Table 2). In the examination of risk factors by multiple logistic regression analysis, high BMI and oral dabigatran were associated with odds ratios for exfoliative esophagitis of 1.139 (*P* = .045) and 10.301 (*P* = .007), respectively (Table 3). Multiple logistic regression analysis with BMI and anticoagulants as independent variables and exfoliative esophagitis as the dependent variable showed a *P* value of .709 for the interaction, which was not significant (data not shown).

**Table 2:**
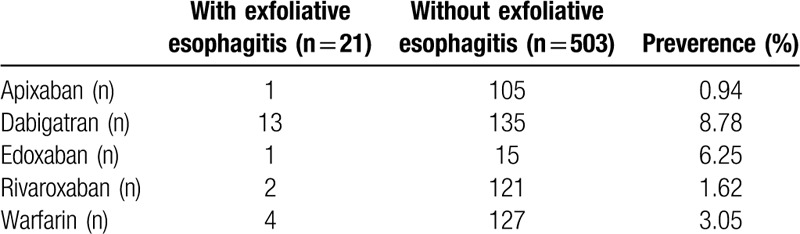
The breakdown of the anticoagulants with or without exfolative esophagitis.

**Table 3:**
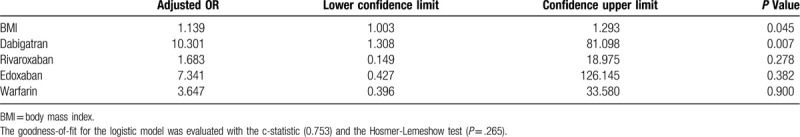
Odds ratio of each factor based on a multivariable logistic regression analysis after variable adjustment.

## 4 Discussion

In the present study, we demonstrated that higher BMI and oral dabigatran were associated with significantly higher risks for exfoliative esophagitis in AF patients. This is the first study to report that obesity is a risk factor for exfoliative esophagitis. Obesity is thought to cause delayed esophageal clearance due to increased abdominal pressure or abnormalities of esophageal peristalsis.^[15]^ Daigneault et al reported that 51% of patients with a mean BMI of 46 had esophageal dysmotility regardless of gastrointestinal symptoms.^[16]^ In fact, our study demonstrated that endoscopic findings did not reveal significant differences in the prevalence of hiatal hernia and reflux esophagitis between patients with and without exfoliative esophagitis. Although esophageal transit time was not measured in this study, esophageal dysmotility may contribute to drug stagnation, resulting in the development of exfoliative esophagitis in obese patients.

Organic factors involved in esophageal stagnation include esophageal compression by an enlarged heart, esophageal achalasia, esophageal cancer, postoperative anastomotic stenosis, and esophageal hiatal hernia.^[10]^ However, in the present study, left atrial size, cardiac performance, and esophageal herniation were not associated with the prevalence of exfoliative esophagitis.

DOACs are becoming increasingly used due to their convenience, but are known to be associated with a higher prevalence of gastrointestinal mucosal injury than warfarin.^[17]^ Dabigatran is the first DOAC launched in Japan and cases of esophagitis and esophageal ulcers caused by dabigatran are increasingly being reported.^[9,11]^ A recent study in Japan reported that dabigatran-induced esophagitis was found in approximately 20% of patients;^[8]^ however, no comparisons with other anticoagulants and subsequent esophageal mucosal injury have been reported. In this study, the prevalence of exfoliative esophagitis was the highest in those taking dabigatran (8.8%), having the only statistically significant result when comparing with other DOACs. Dabigatran is prescribed as a relatively large capsule (18–19 mm in length, 280–390 mg in weight), which can easily lodge in the esophageal tract. Hey et al^[18]^ reported that the size and weight affect the esophageal transit time and that capsules are more likely to adhere to the esophageal mucosa than tablets. Considering these facts, dabigatran has a tendency to stagnate in the esophagus. It has also been considered to cause injury due to an eluting tartaric acid core, which is present only in dabigatran.^[11,16]^ Because dabigatran can be soluble in a strongly acidic environment, tartaric acid is used as the core and coated with dabigatran etexilate. The pH of tartaric acid is 2.4 and its solubility creates a significantly acidic environment. Instructing patients to take dabigatran with a large amount of water, with food, and to maintain an upright position for a while after taking it has been reported as an effective way to avoid dabigatran lodging in the esophagus.^[11]^ We have also encountered cases of exfoliative esophagitis that resolved after telling patients to follow the instructions that taking the drug with plenty of water during a meal and maintain sitting position.^[19]^ In daily practice, although Proton pump inhibitor (PPI) is often administered as a treatment of dabigatran-induced exfoliative esophagitis, it may be invalid given the mechanisms above. Indeed, no difference in PPI administration in dabigatran users with or without esophagitis was reported.^[8]^ Another DOAC, edoxaban, showed the second highest prevalence of exfoliative esophagitis; however, this result may not be robust because the total sample number was small compared with the other anticoagulants.

There are some limitations in this study. First, the patients were those who received catheter ablation, so we cannot rule out the potential effects of the procedure itself. Catheter ablation has been reported to cause injury to the surrounding plexuses by causing changes in esophageal temperature.^[20,21]^ It has also been associated with increased incidences of esophageal spasm, gastrointestinal dysmotility, and gastroesophageal reflux, which may result in a reversible delay in gastrointestinal clearance and the lodging of medications in the gastrointestinal tract.^[22]^ Second, this is the retrospective study at a single center and the sample number is not large. Prospective, large-scale, and multicenter studies are required to clarify more details about DOAC-induced esophagitis.

In conclusion, obesity and oral dabigatran were found to be significant risk factors for the development of exfoliative esophagitis following catheter ablation in patients with AF.

## Author contributions

Hiroki Tajima: Wrote the paper. Acquired and analyzed the data.

Hideo Suzuki: Revised the manuscript and gave final approval of the version to be published.

Kazushi Maruo: Provided support during the statistical analysis.

Toshiaki Narasaka, Daisuke Akutsu, Hirofumi Matsui, Hiro Yamasaki and Yuji Mizokami: Revised the manuscript and gave final approval of the version to be published.

**Conceptualization:** Toshiaki Narasaka, Daisuke Akutsu, Yuji Mizokami.

**Data curation:** Hiroki Tajima.

**Formal analysis:** Hiroki Tajima, Kazushi Maruo.

**Investigation:** Hiroki Tajima.

**Methodology:** Hiro Yamasaki.

**Supervision:** Toshiaki Narasaka, Daisuke Akutsu, Hideo Suzuki, Hiro Yamasaki, Yuji Mizokami.

**Writing – original draft:** Hiroki Tajima.

**Writing – review & editing:** Toshiaki Narasaka, Daisuke Akutsu, Hideo Suzuki, Hirofumi Matsui, Kazushi Maruo, Hiro Yamasaki, Yuji Mizokami.
